# Fear Priming: A Method for Examining Postural Strategies Associated With Fear of Falling

**DOI:** 10.3389/fnagi.2020.00241

**Published:** 2020-08-05

**Authors:** Alexander Stamenkovic, Susanne M. van der Veen, James S. Thomas

**Affiliations:** ^1^Motor Control Laboratory, Department of Physical Therapy, Virginia Commonwealth University, Richmond, VA, United States; ^2^Department of Physical Medicine and Rehabilitation, Virginia Commonwealth University, Richmond, VA, United States

**Keywords:** fear of falling, posture, balance, fear priming, aging

## Abstract

Fear of falling influences postural strategies used for balance, and is key in the maintenance of independent living and quality of life as adults age. However, there is a distinct need for methodology that aims to specifically address and prime fear under dynamic conditions, and to better determine the role of fear in movement preparation. This preliminary study investigated how fear priming influences fear of falling in young and older individuals, and assessed how changes in fear of falling map to movement behavior. Young (21.5 ± 1.7 years, *n* = 10) and older (58.1 ± 2.2 years) participants matched for height, weight, and sex were repeatedly exposed to four different and incrementally challenging laboratory-based slipping perturbations during a self-initiated, goal-directed step and reach task. Both younger and older cohorts showed similar heightened perceptions in fear of falling after fear priming, and changes in peak joint excursions including reduced ankle flexion, and increased lumbar flexion after fear priming. Age-related changes were only evident in total mediolateral center of mass displacement, with younger participants showing greater displacement after fear priming. Despite clear differences in preparatory muscle onsets relative to reach onset seen in older participants, muscle timings or co-contraction indices were not significantly different. Methods utilizing repeated exposure to varying increases of a slip-based postural challenge can successfully prime fear of falling in individuals, regardless of age.

## Introduction

Fear of falling and movement anxiety related to postural threat have been shown to influence postural strategies used for balance, and are key factors in the maintenance of independent living and quality of life as adults age (Young and Williams, 2015). Generally, mechanisms underlying the role of fear in contributing to falls and stability are examined through two main experimental paradigms; using changes in height to induce fear (Adkin et al., 2002; Cleworth et al., 2012, 2016; Zaback et al., 2016), and by manipulating the expectation of an externally produced perturbation (Johnson et al., 2019). From these studies, a “stiffening strategy” characterized by increases in muscle co-activation (Nagai et al., 2012) and reduced ranges of motion and velocity of movement across joints of the lower limb (Brown et al., 2002) is associated with increased postural threat, with the goal of minimizing disturbances to the center of mass (CoM) position (Carpenter et al., 2001). Considering that the majority of falls occur during gait-based events including weight bearing transitions and single-limb support, it is less clear how fear of falling influences the interplay between postural-based movement and task-based movement goals, as previous paradigms are predicated on feedback-based reactive mechanisms that aim to reduce postural changes. In fact, a number of activities, including gait and whole-body reaching, require preparatory postural adjustments across the lower limbs and trunk that drive displacement of the CoM for successful task performance (Brenière et al., 1987; Stapley et al., 1999; Leonard et al., 2009; Stamenkovic and Stapley, 2016).

When examined in the context of volitional movement control, fear of falling shows similar alterations in movement behaviors despite these differences in task goals. Additionally, fear of falling manifests in an impaired and “cautious” gait strategy (Delbaere et al., 2009) yet, it is unknown whether this is solely based on fear components or is multi-dimensional in nature as comparisons are often made between individuals exhibiting low or high fear of falling (Adkin et al., 2002; Nagai et al., 2012; Uemura et al., 2012). Furthermore, these paradigms adopt voluntary movements toward postures affording less stability (e.g., rise to toes – Adkin et al., 2002; Zaback et al., 2016, leg raises –Yiou et al., 2011; Gendre et al., 2016). These findings indicate that fear of falling can be characterized by a conscious prioritization of posture and minimization in CoM displacement. When we also consider age-related declines in postural control, either through the structural degradation of independent sensory processes (Manini et al., 2013) or multi-sensorimotor integration (Papegaaij et al., 2014), which may present as early as middle-age (Humes, 2015), methodology that incorporates increasing fear perceptions and examines movement preparation can provide insights into how fear influences the state of the sensorimotor system. As such, there is a distinct need for methodology that specifically addresses whether fear of falling perceptions can influence movement preparation and planning. Rather than focus on compensatory strategies to the application of an unexpected surface condition across high and low fear populations, the current paradigm aimed to investigate how priming fear (using known changes to surface condition) alters age-related preparatory movement strategies in an initially non-fearful population.

It was hypothesized that repeated exposure to varying degrees of a slipping perturbation would (1) prime state-specific fear in individuals and (2) alter age-related preparatory strategies that would prioritize balance maintenance, including minimizing CoM displacement through increased co-contraction and earlier activation of postural muscle activity relative to movement onset.

## Methods

### Participants

Ten healthy young (5 female; mean age: 21.5 ± 1.7 years; mean height: 1.71 ± 0.07 m; mean weight 69.4 ± 10.3 kg) and 10 anthropometrically matched older participants (5 female; mean age: 58.1 ± 2.2 years; mean height: 1.69 ± 0.09 m; mean weight 69.6 ± 9.12 kg), without any known neurological, visual, or orthopedic impairments gave informed consent for all experimental procedures. Local institutional ethical approval (IRB #13F014) was granted for all protocols and procedures. Assessment of initial perceptions surrounding balance confidence (Activities-Specific Balance Confidence scale – ABC), and fear of falling (via the Falls Efficacy scale – FES) found that participants in both cohorts reported having high balance confidence (ABC: YOUNG, 96.2% + 5.1% vs. OLDER, 91.1% + 6.4%) and low fear of falling (FES: YOUNG, 11.0% + 1.6% vs. OLDER, 11.6% + 3.5%). These were outside of mean scores generally associated with a greater predicted risk of falling (e.g., <67% for ABC – Lajoie and Gallagher, 2004).

### Experimental Apparatus and Set-Up

**FIGURE 1:**
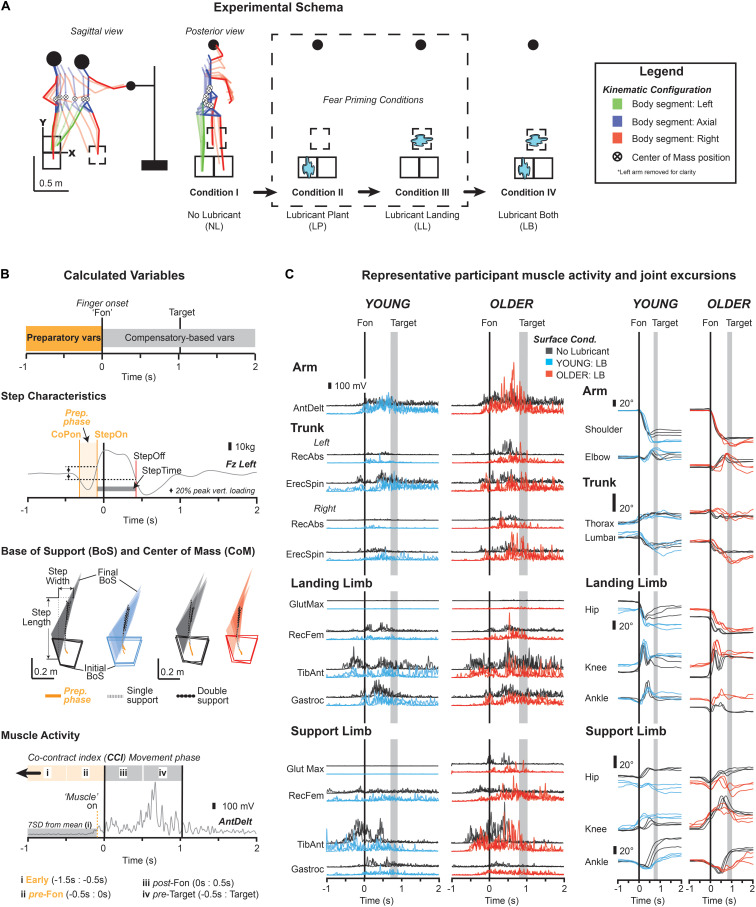
Schematic of fear priming used in the current experimental protocol **(A)**, with description of calculated movement preparation (orange) and compensatory-based variables **(B)**, and representative examples of muscle activity, and joint excursions **(C)**. **(A)** Participants began in a control condition without any slipping perturbation (Condition I – NL) and progressed through to Condition IV (LB) where slipping perturbation was present under both the supporting limb (“plant”) and within the step landing position. **(B)** Calculated variables of interest derived from ground reaction forces (i.e., step characteristics), kinematics (center of mass, CoM), and muscle activity (i.e., muscle onsets and co-contraction indexes, CCI). Variables associated with movement preparation are termed preparatory and highlighted in orange. **(C)** Representative muscle activity and joint excursions prior to movement onset (i.e., Fon), and during the step-and-reach task. Control trials (i.e., Condition I, NL) are depicted in black, with perturbation trials (i.e., Condition IV – LB) for YOUNG and OLDER participants in blue and red, respectively.

Participants stood barefoot on two linoleum covered tri-axial force plates (Model #4060, Bertec, Columbus, OH, United States) that recorded ground reaction forces and moments at 1,000 Hz (Figure 1A). Whole-body kinematics were recorded using a custom designed marker set and 10-camera Vicon Nero system (Vicon, Oxford, United Kingdom) at 100 Hz. Muscle activity for the trunk, lower limb and reaching arm were recorded using a 16-channel Trigno wireless surface electromyography (EMG) system (Delsys, Boston, MA, United States) at 2,000 Hz. EMG set-up, including skin preparation followed procedures set forth in the Surface ElectroMyoGraphy for the Non-Invasive Assessment of Muscles guidelines (SENIAM; Hermens et al., 2000), while surface electrode (Trace1, Nikomed, Hatboro, PA, United States) placement aligned with SENIAM guidelines (Hermens et al., 2000), and previously identified sites for trunk musculature (Stamenkovic and Stapley, 2016). Kinematics, analog EMG, force place and target contact were synchronized through MotionMonitor (Innovative Sports, Chicago, IL, United States). Data collection occurred for a total of 8 s.

### Experimental Procedures

Participants were instructed to reach with their right hand “as fast as possible” while taking a step with their right foot, to complete a whole-body point-to-point movement. Movements were self-initiated with reach onset and offset determined using an infrared sensing system (FRK-030-M-Q8, Ramco Innovations, Des Moines, IA, United States) to identify initiation of hand movement, and contact with the target. Step and reach movements were made under 4 different surface-lubricating conditions created to elicit a greater likelihood of perturbation (e.g., through slipping and falling). The four conditions included no lubricant (NL), lubricant under the left planted leg (LP), under the right leg landing area (LL), and a combination of the prior two conditions, affecting both the postural and moving limbs (LB). Target position was based on standardized measures of hip height (from ground), trunk length, and arm length to produce a vertical target position that required 15° of trunk flexion (Thomas et al., 2008) with the anterior-posterior distance of the target increased by an additional 50% of total hip height to ensure a step was required (Figure 1A). Trials (*n* = 3) for each perturbation condition were presented in a blocked fashion (NL, LP, LL, LB) with the intent to provide progressively greater challenge under which reaching movements were made and heighten overall perceptions of fall risk. Participants were familiarized with the task and speed necessary in a short practice session prior to data collection.

### Data Analysis

Analysis was completed offline using Matlab (ver. R2018, The Mathworks, Natick, MA, United States). Kinematic data were smoothed using a 40-point Savitzky–Golay filter and DC offset removed. Analog EMG signals were amplified (1,000×), notch filtered (60 Hz) before being de-meaned, rectified and low-pass filtered using a 4th order zero-lag Butterworth at 100 Hz. EMG was normalized to the maximum amplitude recorded across all conditions per individual and muscle.

Prior to each condition, two 100 mm Visual Analog scales (VASs) were used to assess state-specific fear of falling, regarding expectations of participant’s “likelihood of falling” and “concern of falling.” Figure 1B highlights step and CoM based characteristics, and temporal and amplitude-based muscle activity related to movement preparation (Figure 1B: orange). Preparatory anteroposterior (AP) and mediolateral (ML) CoM acceleration and excursion measures were determined using the onset of center of pressure (Figure 1B: CoPon), and landing limb unloading (i.e., step onset, Figure 1B: StepOn). Further, timing of trunk and lower limb muscle activity were calculated relative to the initiation of reach. Muscle onsets were determined using a custom algorithm based on the maintenance of muscle activity for 50 ms beyond 7 SD of average baseline activity and confirmed via visual inspection (Teasdale et al., 1993). Measures of muscle amplitude were determined using the ratio of agonist to antagonist activation, or co-contraction index (CCI; Craig et al., 2016). The CCI was divided into four distinct phases; (i) early activity 1.5 to 0.5 s prior to reach onset, (ii) preparatory activity 0.5 s prior to reach onset, (iii) movement activity 0.5 s following reach onset, and (iv) termination activity 0.5 s prior to target contact.

To characterize how changes in fear of falling mapped to movement behavior changes in step characteristics including AP distance between malleoli of the support and landing limb (StepLength), ML distance between malleoli of the support and landing limb (StepWidth), and time between step onset and offset (StepTime), as well as peak-to-peak joint and CoM excursions across the duration of movement were also analyzed (Figure 1C).

### Statistical Analysis

The effect of fear on movement behaviors were assessed using separate two-way mixed repeated measures ANOVAs (Condition × AgeGroup) using SPSS (version 25, IBM, Armonk, NY, United States). To address our primary outcomes, repeated measures ANOVAs on changes in state-specific fear (Dependent variables, DV: VAS “Likely to fall,” VAS “Concerned about falling”), were followed by repeated measures ANOVAs for preparatory variables including step characteristics (DV: CoPon, StepOn), CoM characteristics (DV: preparatory CoM AP/ML accelerations and excursions), muscle onsets (DV: see muscle list), and co-contraction indices (DV: CCI i, CCI ii). Secondary analyses investigated additional movement behavior metrics including compensatory changes in step characteristics (DV: StepOff, StepTime, StepLength, StepWidth), CoM excursions (DV: CoM total AP excursion, CoM total ML excursion), muscle co-contraction indices (DV: CCI iii, CCI iv), and peak joint excursions (DV: ankle, knee, hip, lumbar, thorax, shoulder, elbow) associated with fear of falling. Bonferroni–Holm adjustments were applied to main repeated measures ANOVA results to reduce the family-wise error rate before determining significance across related measures (i.e., step or CoM characteristics, muscle onsets, CCI, joint excursions). This was achieved by altering the initial level of significance (*p* < 0.05) with respect to the total number of tests performed to produce a more conservative significance level such that; *p* = 0.05/(# DVs × 2 main effect/interactions). Greenhouse–Geisser adjustments were made in cases where violations of sphericity were observed. When applicable, further *post hoc* analyses were conducted with Bonferroni adjustment for multiple pairwise comparisons.

## Results

Descriptive statistics and results from individual repeated measures ANOVAs are available in tabular form (see Supplementary Material).

### Influence of Fear Priming on State-Specific Fear of Falling

**FIGURE 2:**
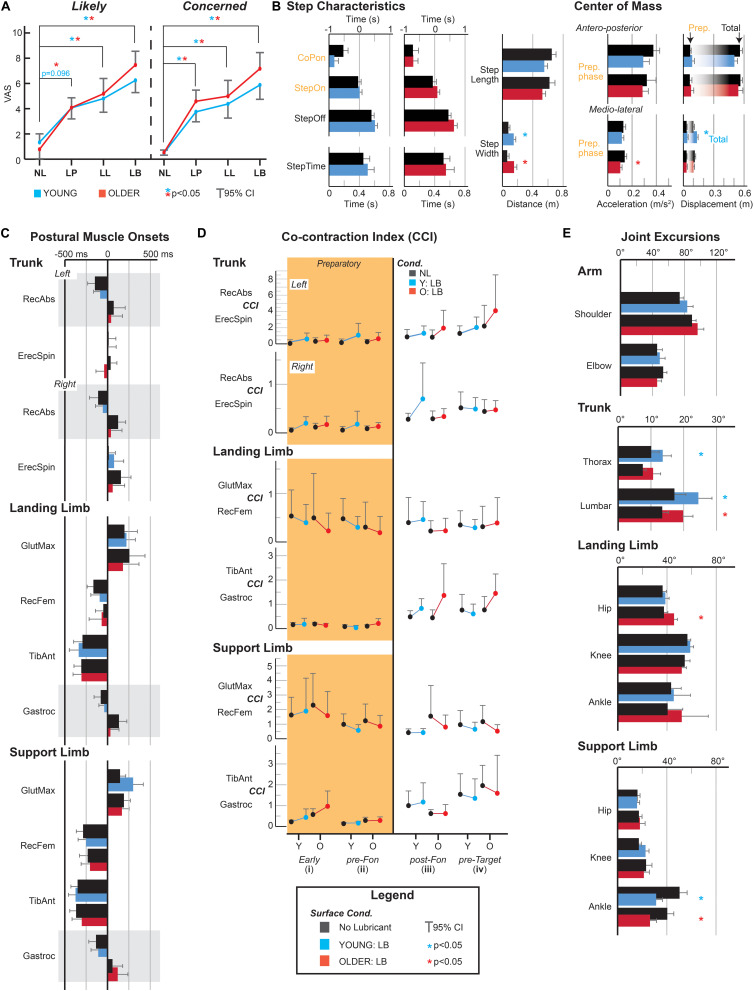
Comparison of fear cognitions **(A)**, step characteristics and center of mass measures **(B)**, muscle onsets **(C)**, muscle co-contraction indexes (CCI) **(D)**, and joint excursions **(E)** across fear conditions in YOUNG (blue) and OLDER (red) participants. **(A)** Visual Analog scale (VAS) outcomes on self-reported perceptions on the likelihood of falling (“Likely”) and concern about falling (“Concerned”) revealed an incremental increase in fear cognitions across fear priming conditions regardless of age group. **(B)** Step-based and center of mass (CoM) movement characteristics revealed differential changes on preparatory and compensatory movement behaviors. **(C)** Muscle onsets for postural muscles of the trunk and lower limbs. While onsets did not significantly vary across surface condition or age, a number of muscles (shown in gray) showed delayed activations following movement onset in OLDER participants. **(D)** Muscle CCI across preparatory (i, ii – orange) and compensatory movement phases (iii, iv). No significant differences in CCI measures were seen across surface condition or age. **(E)** Peak-to-peak joint excursions for the reaching arm, trunk, and lower limbs. Lumbar excursions were increased and, ankle excursions in the supporting limb were decreased in both cohorts as a result of surface condition. All values reported as mean + 95% confidence interval. *p < 0.005 (Bonferroni–Holm adjusted).

Figure 2A shows the changes in fear perceptions regarding falling as participants made step and reach movements across increasingly challenging surface conditions. A main effect of surface condition was seen for both VAS regarding state-specific expectations about falling [“Likely”: *F*_(__1.845,29.518__)_ = 36.463, *p* < 0.001, η^2^*_*p*_* = 0.695; “Concerned”: *F*_(__3,48__)_ = 31.343, *p* < 0.001, η^2^*_*p*_* = 0.662]. *Post hoc* analyses revealed that both YOUNG and OLDER cohorts had heightened perceptions of falling in the LB surface condition relative to the control (NL) condition (“Likely” – YOUNG: *p* = 0.015; OLDER: *p* = 0.001; “Concerned” – YOUNG: *p* = 0.007; OLDER: *p* = 0.004).

### Characterizing Movement Behavior: Movement Preparation

#### Stepping and CoM Characteristics

Figure 2B shows changes in stepping and CoM characteristics prior to, and following movement onset. Variables highlighted in orange represent those that were preparatory in nature (for visual representation see Figure 1B). Following Bonferroni–Holm adjustments, only CoM acceleration during the preparatory phase showed a significant main effect of surface condition [*F*_(__1,18__)_ = 11.06, *p* = 0.004, η^2^*_*p*_* = 0.381].

#### Muscle Activity

Clear preparatory activity was seen in the trunk and lower limbs, particularly within the erector spinae and tibialis anterior (see Figure 1C for representative example). When pooled, analyses revealed no differences in muscle onsets (Figure 2C) or preparatory amplitude measures (i.e., CCI) in the “early” and “preparatory” movement phases (see Figure 2D, orange background). However, despite a lack of differences in muscle onsets, a clear distinction in the onset of certain muscles was seen between age groups. Specifically, YOUNG participants displayed bilateral activity of rectus abdominis and gastrocnemius that preceded movement onset, while these muscles often activated following the initiation of reach in OLDER participants (Figure 2C, gray boxes). Surprisingly, these timings did not alter as a consequence of condition.

### Characterizing Movement Behavior: Compensatory Strategies

#### Stepping and CoM Characteristics

A number of step and CoM measures showed differences following movement onset (Figure 2B). Of note, an interaction effect was present for total ML CoM excursion [*F*_(__1,18__)_ = 11.630, *p* = 0.003, η^2^*_*p*_* = 0.392]. *Post hoc* analysis revealed that this was driven by greater excursion in YOUNG participants (*p* = 0.001). By extension, step width showed a main effect of surface condition [*F*_(__1,18__)_ = 19.020, *p* < 0.001, η^2^*_*p*_* = 0.514], increasing in both YOUNG and OLDER participants.

#### Muscle Activity

Analysis of CCI during compensatory phases of movement (i.e., Figure 2D, CCI iii, CCI iv) did not show any differences across surface condition or age group.

#### Joint Excursions

Changes in surface condition influenced peak to peak joint kinematics (see Figure 1C for representative example and Figure 2E for pooled means), resulting in similar increases in lumbar flexion [*F*_(__1,18__)_ = 23.74, *p* < 0.001, η^2^*_*p*_* = 0.597], and reductions in support limb ankle flexion [*F*_(__1,18__)_ = 22.56, *p* < 0.001, η^2^*_*p*_* = 0.585] across age groups respectively. Greater thoracic flexion [*F*_(__1,18__)_ = 10.77, *p* = 0.005, η^2^*_*p*_* = 0.402] and leading limb hip flexion [*F*_(__1,18__)_ = 10.29, *p* = 0.005, η^2^*_*p*_* = 0.391] was seen as a consequence of surface condition.

## Discussion

The present study examined the ability of a perturbation-based step and reach paradigm to elicit fear and characterize how such fear cognitions shape movement preparation behavior. By gradually exposing participants to changes in surface condition, the paradigm was successfully able to produce increases in perceived fear of falling (Figure 2A). The increases in state-specific fear in the current study lie in contrast to previous findings, where unpredictable postural perturbations initially increase anxiety, which significantly reduces following repeated exposure (Johnson et al., 2019). However, it is unclear whether this is related to the type of perturbation produced. Exposure to slipping-specific perturbations have been associated with recovery strategies that adapt at similar rates to result in fewer falls between younger and older adults, although fear was not recorded in these studies (Pavol et al., 2002; Pai et al., 2007). By producing perceptions of fear of falling in a cohort of initially non-fearful younger and older adults, the current paradigm also induced changes in compensatory stepping strategies and peak joint excursions. Specifically, OLDER participants showed a reduction in total ML CoM excursion with shorter and wider steps that followed (rather than preceded) movement of the reaching arm during the more fearful LB condition. In fact, evidence of an attempt to minimize ML CoM excursion (rather than increase, as seen in the YOUNG cohort) supports the adoption of a balance-centric postural strategy, as lateral instability is associated with an increased fall risk in older adults (Rogers and Mille, 2003). Similar to expectations from previous feedback-based paradigms assessing fear of falling, both YOUNG and OLDER participants displayed reductions in ankle excursion indicative of the adoption of a stiffening strategy. Considering the lack of alterations in arm kinematics, the greater excursions of the trunk that complemented changes in ankle excursion are most likely a consequence of motor equivalence in order to successfully achieve task goals (i.e., target contact). Whether this attribute of the current protocol can be leveraged in the future assessment of the link between fear cognitions and balance performance is unclear, however both step characteristics and trunk kinematics are shown to be important predictors of falls and recovery step after lab-induced trips, and highly implicated in falls risk (Marone et al., 2011).

Despite changes in fear influencing step characteristics and joint excursions across the movement, similar alterations were not reflected in measures of underlying muscle activity. While age-related differences in muscle onsets showed delays in the production of older adults preparatory postural adjustments, these were not affected by fear priming. Further, the impact of fear priming was not observable in the timing or muscle co-contraction index (i.e., CCI) across the trunk and lower limbs. Considering that increases in co-contraction are often found when examining fear-related changes that occur with postural control (Nagai et al., 2012) it is surprising that these were not replicated in the current study, especially during preparatory periods preceding movement onset. As all conditions required a step to achieve the task, it is plausible that increases in co-contraction, especially around the ankle joint would have impeded task completion by making it more difficult to initiate a step. Therefore, a generalized movement preparation strategy was adopted, despite greater concerns regarding the likelihood of falling. Additionally, the discrepancy between falls efficacy (via FES) and state-specific fear of falling (via VAS) may provide a conceptual explanation to the broad lack of changes in preparatory strategies seen in the current study. Hadjistavropoulos et al. (2011) argue that both are separate constructs that contribute to fall outcomes and impact on balance performance. Therefore, a combination of high efficacy in one’s ability to maintain balance, and task requirements (i.e., promoting movement or maintaining balance) may mediate changes to preparatory postural alterations that would often follow increases in high contextual anxiety and fear of falling, particularly if concerns regarding falling align with the situational likelihood of a fall occurring (i.e., that an individual accurately appraises the “current” situation). In fact, the mechanism underlying this interaction is unclear, with work investigating neural correlates responsible for the specific interaction of fear of falling on movement behavior limited. As stiffening strategies adopted in static perturbations and cautious gait are thought to represent a shift to a conscious and cortical-based control of posture, frontoparietal interactions provide a viable conduit for investigation. In fact, decision confidence (Bang and Fleming, 2018), fear conditioning and extinction (Giustino and Maren, 2015), and executive behavior planning (particularly with respect to the future consequences of actions – Matsumoto and Tanaka, 2004) have all been implicated with medial pre-frontal cortex function. Only a single recent investigation has provided evidence that individuals with generalized high fear of falling have greater neural inefficiency (i.e., higher brain activations associated with decrements in performance) in the pre-frontal cortex, however this occurs when a concomitant cognitive process (i.e., dual-task) is applied (Holtzer et al., 2019). Whether this applies purely to fear perceptions without confounding factors is unknown, however future examinations could leverage the current paradigm to parse out such influences on movement preparation, especially considering that planning actions between predictable and unpredictable events are thought to have distinct neural substrates within the pre-frontal cortex (Koechlin et al., 2000).

### Study Limitations

While the possible effects of condition order cannot be discounted, perturbation conditions were deliberately presented in a blocked fashion with the intent to provide progressively greater challenge under which reaching movements were made, thus heightening overall perceptions of fall risk. This was based on a similar framework upon which graded exposure paradigms operate (Trost et al., 2009). While this makes it challenging to parse motor learning effects from those of fear of falling, we would consider that fear conditioning would be modulated (i.e., extinguished) by improvements based on motor learning and knowledge of continued task success, rather that the maintenance (and general increase of fear perceptions) seen in the current study.

Further, as general safety and feasibility of the paradigm was a key consideration in this preliminary study we deliberately recruited OLDER adults between 55 and 65 which is relatively young compared to the literature (i.e., 65+ years old). However, our differences in CoM excursion and preparatory sequences of muscle activity highlight that changes in movement behavior are detectable even within a middle-aged cohort. Considering the focus on older cohorts (65+ years old) within the literature, this highlights the importance of emphasizing the investigation of a greater spectrum of age across human development, especially in the context of understanding mechanisms surrounding fear of falling influences on postural control (Hadjistavropoulos et al., 2011).

## Conclusion

The current paradigm shows that fear priming, and repeated exposure to progressively increasing perturbations that challenge balance, have the capacity to increase fear of falling in an initially non-fearful cohort of younger and older adults. As such, this study provides a novel assessment of how fear conditioning changes motor behavior in a healthy cohort and provides a method to examine how fear changes motor planning in individuals.

## Data Availability Statement

The datasets generated for this study are available on request to the corresponding author.

## Ethics Statement

The studies involving human participants were reviewed and approved by the Ohio University Institutional Review Board (IRB #13F014). The patients/participants provided their written informed consent to participate in this study.

## Author Contributions

JT conceived and designed the study, and collected the data. AS and SV performed the analysis. AS produced the first draft of the manuscript. All authors contributed to the interpretation of data, critical review of the manuscript, and approval of the final version of this manuscript.

## Conflict of Interest

The authors declare that the research was conducted in the absence of any commercial or financial relationships that could be construed as a potential conflict of interest.

## Abbreviations

ABC: Activities of Balance Confidence scale
BoS: base of support
CCI: co-contraction index
CoM: center of mass
FES: Falls Efficacy scale
LB.: “lubricant both” perturbation condition
LL: “lubricant landing” perturbation condition
LP: “lubricant plant” perturbation condition
NL: “no lubricant” perturbation condition
VAS: Visual Analog scale.
