# Supplementary material for: Fear Priming: A Method for Examining Postural Strategies Associated With Fear of Falling
Source: Front Aging Neurosci. 2020 Aug 5;12:241. doi: 10.3389/fnagi.2020.00241 (PMC7419599; doi:10.3389/fnagi.2020.00241)
Supplement: Supplementary file 1 [file Table_1.pdf]

Stamenkovic et al., 2020

Fear priming: a method for examining postural strategies associated with fear of falling

Supplementary Materials

Number of Tables: 6

Table 1: VAS descriptive statistics and repeated measures ANOVA results

	NL				LP				LL				LB							
	YOUNG		OLDER		YOUNG		OLDER		YOUNG		OLDER		YOUNG		OLDER					
	$\bar{x}$	s	$\bar{x}$	s	$\bar{x}$	s	$\bar{x}$	s	$\bar{x}$	s	$\bar{x}$	s	$\bar{x}$	s	$\bar{x}$	s		$F_{(3,48)}$	*p*	η^2_p
Likely (mm)	1.48	2.87	0.73	0.69	4.43	2.93	3.88	1.76	5.16	3.30	5.38	3.37	6.21	3.27	7.64	2.91	*Cond.*	36.463†	<0.001*	0.695
																	*Cond.**Age*	1.49†	0.229	0.085
Concerned (mm)	0.60	0.54	0.55	0.49	3.74	2.53	4.32	2.35	4.39	3.34	5.13	3.55	5.63	3.25	7.23	3.73	*Cond.*	31.34	<0.001*	0.662
																	*Cond.**Age*	0.59	0.623	0.036

*$p < 0.05$ (Bonferroni-Holm adj.)

†$F_{(1.845,29.518)}$

Table 2: Step characteristics descriptive statistics and repeated measures ANOVA results

	NL				LB							
	YOUNG		OLDER		YOUNG		OLDER					
	$\bar{x}$	s	$\bar{x}$	s	$\bar{x}$	s	$\bar{x}$	s		$F_{(1,18)}$	*p*	η^2_p
CoPon[a] (ms)	-525	423	-716	505	-827	377	-705	423	*Cond.*	3.20	0.090	0.151
									*Cond.*Age*	3.72	0.070	0.171
StepOn[a] (ms)	-43	204	-62	279	6	205	87	207	*Cond.*	4.78	0.042	0.210
									*Cond.*Age*	0.28	0.283	0.064
StepOff[b] (ms)	406	182	453	194	513	194	634	273	*Cond.*	8.34	0.010	0.317
									*Cond.*Age*	0.56	0.463	0.030
StepLength[b] (cm)	64.99	13.53	62.02	16.84	55.11	10.43	52.64	11.47	*Cond.*	10.01	0.005	0.357
									*Cond.*Age*	0.01	0.936	0.001
StepWidth[b] (cm)	16.89	4.85	15.55	5.83	23.04	6.23	23.43	8.78	*Cond.*	19.02	***<0.001****	0.514
									*Cond.*Age*	0.29	0.598	0.016
StepTime[b] (s)	0.45	0.19	0.52	0.22	0.51	0.23	0.55	0.29	*Cond.*	1.16	0.297	0.060
									*Cond.*Age*	0.10	0.760	0.005

[a]Preparatory variables
[b]Compensatory variables

*p < 0.05 (Bonferroni-Holm adj.)

Table 3: Center of mass descriptive statistics and repeated measures ANOVA results

	**NL**				**LB**							
	YOUNG		OLDER		YOUNG		OLDER					
	$\bar{x}$	s	$\bar{x}$	s	$\bar{x}$	s	$\bar{x}$	s		**$F_{(1,18)}$**	***p***	**η^2_p**
AP												
Prep. Acc[a]	37.49	13.29	32.10	20.45	29.17	13.38	28.58	12.83	*Cond.*	6.83	0.018	0.005
(cm/s²)									*Cond.*Age*	1.13	0.302	0.059
Prep. Exc[a]	6.76	2.65	6.42	5.50	8.75	5.86	7.67	5.66	*Cond.*	2.73	0.116	0.132
(cm)									*Cond.*Age*	0.14	0.713	0.008
Tot. Exc[b]	55.91	6.55	56.39	5.31	50.40	8.73	54.29	11.78	*Cond.*	4.15	0.057	0.187
(cm)									*Cond.*Age*	0.83	0.374	0.044
ML												
Prep. Acc[a]	12.79	4.96	13.91	4.51	11.56	4.52	10.27	4.05	*Cond.*	11.06	**_0.004*_**	0.381
(cm/s²)									*Cond.*Age*	2.71	0.117	0.131
Prep. Exc[a]	3.24	1.33	3.32	1.36	3.94	2.13	3.20	2.03	*Cond.*	0.96	0.340	0.051
(cm)									*Cond.*Age*	1.92	0.183	0.096
Tot. Exc[b]	10.04	2.83	11.22	2.89	13.45	3.14	10.69	2.88	*Cond.*	6.24	**_0.022*_**	0.257
(cm)									*Cond.*Age*	11.63	**_0.003*_**	0.392

[a]Preparatory variables

[b]Compensatory variables

*p < 0.05 (Bonferroni-Holm adj.)

Table 4: Muscle onset descriptive statistics and repeated measures ANOVA results

	NL				LB							
	YOUNG		OLDER		YOUNG		OLDER					
	$\bar{x}$	s	$\bar{x}$	s	$\bar{x}$	s	$\bar{x}$	s		$F_{(1,18)}$	p	η^2_p
Left												
RecAbs[a]	-76	217	152	179	-82	145	40	184	*Cond.*	1.10	0.309	0.065
									*Cond.*Age*	0.90	0.356	0.053
ErecSpin[a]	1	191	169	187	65	279	59	172	*Cond.*	0.16	0.698	0.010
Right									*Cond.*Age*	2.25	0.154	0.123
RecAbs[a]	-135	177	72	239	-106	166	44	161	*Cond.*	0.00	0.990	0.001
									*Cond.*Age*	0.35	0.562	0.021
ErecSpin[a]	0	211	35	90	-18	169	-41	163	*Cond.*	1.35	0.263	0.078
Landing Limb									*Cond.*Age*	0.53	0.476	0.032
GlutMax[a]	237	257	239	305	225	207	192	222	*Cond.*	0.143[#]	0.715	0.018
									*Cond.*Age*	0.048[#]	0.832	0.006
RecFem[a]	-146	140	-50	181	-84	214	-70	239	*Cond.*	0.10	0.752	0.006
									*Cond.*Age*	0.37	0.549	0.023
TibAnt[a]	-292	272	-308	140	-347	292	-313	150	*Cond.*	1.02	0.327	0.060
									*Cond.*Age*	0.76	0.397	0.045
Gastroc[a]	-80	143	133	200	-50	148	33	181	*Cond.*	0.63	0.438	0.038
Support Limb									*Cond.*Age*	2.15	0.163	0.119
GlutMax[a]	137	158	173	158	350	209	121	74	*Cond.*	4.708[#]	0.062	0.370
									*Cond.*Age*	12.73[#]	0.007	0.614
RecFem[a]	-282	174	-237	192	-253	170	-204	149	*Cond.*	0.22	0.643	0.014
									*Cond.*Age*	0.00	0.977	0.001
TibAnt[a]	-364	170	-383	138	-391	153	-330	301	*Cond.*	0.10	0.759	0.006
									*Cond.*Age*	0.99	0.335	0.058
Gastroc[a]	-137	207	59	214	-126	178	116	279	*Cond.*	0.32	0.581	0.019
									*Cond.*Age*	0.15	0.707	0.009

[a]Preparatory variables

[b]Compensatory variables

*$p < 0.05$ (Bonferroni-Holm adj.)

[#]$F_{(1,8)}$

Table 5: Muscle co-contraction index (CCI) descriptive statistics and repeated measures ANOVA results

	NL				LB							
	YOUNG		OLDER		YOUNG		OLDER					
	$\bar{x}$	s	$\bar{x}$	s	$\bar{x}$	s	$\bar{x}$	s		$F_{(1,16)}$	p	η^2_p
					i) Early							
Left												
RecAbs/	0.23	0.36	0.32	0.56	0.64	0.91	0.44	0.82	*Cond.*	6.42	0.022	0.286
ErecSpin[a]									*Cond.*Age*	1.79	0.200	0.100
Right												
RecAbs/	0.06	0.06	0.12	0.10	0.19	0.18	0.17	0.22	*Cond.*	6.19	0.024	0.279
ErecSpin[a]									*Cond.*Age*	1.14	0.302	0.066
Landing Limb												
GlutMax/	0.53	0.70	0.49	1.20	0.40	0.49	0.21	0.50	*Cond.*	2.59	0.127	0.139
RecFem[a]									*Cond.*Age*	0.31	0.587	0.019
TibAnt/	0.16	0.12	0.21	0.07	0.18	0.33	0.13	0.12	*Cond.*	0.56	0.464	0.034
Gastroc[a]									*Cond.*Age*	1.19	0.292	0.069
Support Limb												
GlutMax/	1.60	1.62	2.30	2.83	1.90	2.94	1.56	2.19	*Cond.*	0.20	0.661	0.012
RecFem[a]									*Cond.*Age*	1.08	0.315	0.063
TibAnt/	0.23	0.11	0.56	0.38	0.44	0.52	0.97	0.95	*Cond.*	2.82	0.113	0.150
Gastroc[a]									*Cond.*Age*	0.27	0.614	0.016
					ii) preFon							
Left												
RecAbs/	0.27	0.42	0.26	0.37	1.05	1.94	0.65	0.98	*Cond.*	4.51	0.050	0.220
ErecSpin[a]									*Cond.*Age*	0.50	0.491	0.030
Right												
RecAbs/	0.09	0.09	0.10	0.06	0.20	0.35	0.13	0.10	*Cond.*	1.38	0.257	0.079
ErecSpin[a]									*Cond.*Age*	0.37	0.553	0.022
Landing Limb												
GlutMax/	0.48	0.54	0.30	0.67	0.30	0.29	0.19	0.44	*Cond.*	3.07	0.099	0.161
RecFem[a]									*Cond.*Age*	0.16	0.697	0.010
TibAnt/	0.11	0.07	0.13	0.03	0.09	0.06	0.21	0.26	*Cond.*	0.33	0.571	0.020
Gastroc[a]									*Cond.*Age*	1.25	0.280	0.073
Support Limb												
GlutMax/	0.99	0.93	1.22	1.51	0.57	0.52	0.87	0.97	*Cond.*	3.98	0.063	0.199
RecFem[a]									*Cond.*Age*	0.02	0.878	0.002
TibAnt/	0.16	0.06	0.27	0.22	0.17	0.11	0.29	0.22	*Cond.*	0.06	0.808	0.004
Gastroc[a]									*Cond.*Age*	0.00	0.948	0.001
					iii) postFon							
Left												
RecAbs/	0.85	1.20	0.82	1.17	1.30	1.18	1.94	2.86	*Cond.*	4.23	0.056	0.209
ErecSpin[a]									*Cond.*Age*	0.75	0.401	0.044
Right												
RecAbs/	0.23	0.17	0.29	0.21	0.66	0.96	0.34	0.21	*Cond.*	1.74	0.206	0.098
ErecSpin[a]									*Cond.*Age*	1.14	0.302	0.066
Landing Limb												
GlutMax/	0.40	0.67	0.17	0.28	0.46	0.49	0.18	0.33	*Cond.*	0.11	0.748	0.007
RecFem[a]									*Cond.*Age*	0.04	0.843	0.003
TibAnt/	0.48	0.34	0.45	0.42	0.83	0.52	1.37	1.69	*Cond.*	4.07	0.061	0.203
Gastroc[a]									*Cond.*Age*	0.80	0.384	0.048
Support Limb												
GlutMax/	0.41	0.35	1.54	2.75	0.43	0.35	0.78	1.11	*Cond.*	1.63	0.220	0.092
RecFem[a]									*Cond.*Age*	1.83	0.194	0.103
	1.00	0.92	0.62	0.25	1.16	1.21	0.63	0.55	*Cond.*	0.35	0.565	0.021

TibAnt/ Gastroc[a]									*Cond.*Age*	0.23	0.606	0.017
Left				***iv) preTarget***								
RecAbs/ ErecSpin[a]	1.27	1.24	2.20	3.33	2.02	1.68	4.09	5.76	*Cond.*	5.90	0.027	0.270
									*Cond.*Age*	1.09	0.312	0.064
Right												
RecAbs/ ErecSpin[a]	0.52	0.42	0.44	0.32	0.49	0.30	0.47	0.25	*Cond.*	0.00	0.982	0.001
									*Cond.*Age*	0.18	0.674	0.011
Landing Limb												
GlutMax/ RecFem[a]	0.35	0.38	0.31	0.55	0.29	0.23	0.39	0.68	*Cond.*	0.01	0.941	0.001
									*Cond.*Age*	0.35	0.565	0.021
TibAnt/ Gastroc[a]	0.77	0.84	0.76	0.73	0.60	0.53	1.46	1.03	*Cond.*	1.06	0.320	0.062
									*Cond.*Age*	2.70	0.120	0.144
Support Limb												
GlutMax/ RecFem[a]	0.94	1.29	1.19	1.43	0.65	0.64	0.53	0.58	*Cond.*	6.16	0.025	0.278
									*Cond.*Age*	0.89	0.361	0.052
TibAnt/ Gastroc[a]	1.54	1.28	1.96	1.27	1.35	1.22	1.60	2.36	*Cond.*	0.37	0.550	0.023
									*Cond.*Age*	0.04	0.852	0.002

[a]Preparatory variables

[b]Compensatory variables

*p < 0.05 (Bonferroni-Holm adj.)

Table 6: Joint excursion descriptive statistics and repeated measures ANOVA results

	NL				LB							
	YOUNG		OLDER		YOUNG		OLDER					
	$\bar{x}$	s	$\bar{x}$	s	$\bar{x}$	s	$\bar{x}$	s		$F_{(1,18)}$	*p*	η^2_p
Arm												
Shoulder[b]	75.2	12.0	89.2	12.6	82.9	20.2	96.6	19.8	*Cond.*	6.85	0.019	0.300
									*Cond.*Age*	0.00	0.955	0.001
Elbow[b]	45.8	18.2	53.4	11.0	48.4	18.0	46.2	13.3	*Cond.*	0.37	0.550	0.023
									*Cond.*Age*	1.73	0.206	0.098
Trunk												
Thorax[b]	8.5	2.9	7.5	2.3	12.8	4.8	10.7	5.4	*Cond.*	10.77	***0.005****	0.402
									*Cond.*Age*	0.25	0.623	0.015
Lumbar[b]	14.8	4.8	13.4	4.5	22.3	8.4	19.8	7.3	*Cond.*	23.74	***<0.001****	0.597
									*Cond.*Age*	0.15	0.703	0.009
Landing Limb												
Hip[b]	35.1	7.5	37.5	7.2	37.8	7.6	45.7	6.7	*Cond.*	10.29	***0.005****	0.391
									*Cond.*Age*	2.70	0.120	0.144
Knee[b]	56.3	6.7	54.4	10.5	58.2	5.9	51.9	9.3	*Cond.*	0.02	0.835	0.001
									*Cond.*Age*	0.89	0.360	0.053
Ankle[b]	41.7	12.6	46.4	28.8	43.5	17.8	54.7	29.3	*Cond.*	1.32	0.267	0.076
									*Cond.*Age*	0.34	0.342	0.001
Support Limb												
Hip[b]	16.1	5.8	17.3	6.4	15.3	5.1	18.1	6.6	*Cond.*	0.00	0.991	0.001
									*Cond.*Age*	0.25	0.626	0.015
Knee[b]	16.6	4.5	23.1	11.3	20.9	4.8	21.3	9.3	*Cond.*	0.32	0.580	0.020
									*Cond.*Age*	1.99	0.178	0.110
Ankle[b]	53.5	11.6	40.4	12.2	33.7	13.7	27.4	12.8	*Cond.*	22.56	***<0.001****	0.585
									*Cond.*Age*	0.33	0.332	0.059

[a]Preparatory variables

[b]Compensatory variables

*p < 0.05 (Bonferroni-Holm adj.)
